# A Hierarchical Coarse-Grained (All-Atom-to-All-Residue) Computer Simulation Approach: Self-Assembly of Peptides

**DOI:** 10.1371/journal.pone.0070847

**Published:** 2013-08-13

**Authors:** Ras B. Pandey, Zhifeng Kuang, Barry L. Farmer

**Affiliations:** 1 Department of Physics and Astronomy, University of Southern Mississippi, Hattiesburg, Mississippi, United States of America; 2 Materials and Manufacturing Directorate, Air Force Research Laboratory, Wright Patterson Air Force Base, Dayton, Ohio, United States of America; Russian Academy of Sciences, Institute for Biological Instrumentation, Russian Federation

## Abstract

A hierarchical computational approach (all-atom residue to all-residue peptide) is introduced to study self-organizing structures of peptides as a function of temperature. A simulated residue-residue interaction involving all-atom description, analogous to knowledge-based analysis (with different input), is used as an input to a phenomenological coarse-grained interaction for large scales computer simulations. A set of short peptides P1 (^1^H ^2^S ^3^S ^4^Y ^5^W ^6^Y ^7^A ^8^F ^9^N ^10^N ^11^K ^12^T) is considered as an example to illustrate the utility. We find that peptides assemble rather fast into globular aggregates at low temperatures and disperse as random-coil at high temperatures. The specificity of the mass distribution of the self-assembly depends on the temperature and spatial lengths which are identified from the scaling of the structure factor. Analysis of energy and mobility profiles, gyration radius of peptide, and radial distribution function of the assembly provide insight into the multi-scale (intra- and inter-chain) characteristics. Thermal response of the global assembly with the simulated residue-residue interaction is consistent with that of the knowledge-based analysis despite expected quantitative differences.

## Introduction

Peptides are some of the most versatile constituents in designing advanced materials, from bio-functionalized nanoparticles [1]–[10] to modulating the kinetics of proteins in cells and beyond. Specificity of the amino acids in a short peptide chain is key to their selective binding (covalent and non-covalent) to substrates. Peptides have become a valuable constitutive component in both materials as well as drug design due to prolific conformational response with specific residue interactions. Understanding the unique interaction of a peptide is a challenge in itself. For example, the residue-residue interaction in a solution of free amino acids could be different from a residue-residue interaction in an isolated peptide (intra-chain) or in a peptide melt due to the interplay between the steric constraints of covalent peptide bonds and specific residue interactions. Including solvent, substrate, and other components enhances the complexity in understanding the effect of residue-residue interactions. To probe such systems, one has to start from the building blocks, i.e. amino acids, and develop a feasible method (e.g., bottom-up) to examine the consequences of residue interactions. In this article we introduce such an approach and address how peptides assemble. Since temperature competes directly with the interactions, it is a natural parameter to examine its response in peptide assembly and dispersion.

Residue-residue interaction [11]–[18] is critical in understanding the multi-scale equilibrium structure of large peptides and proteins where it is not feasible to incorporate atomic scale details, a challenging issue in computational modeling. Some degree of coarse-graining and approximations are therefore unavoidable in order to carry out large-scale simulations [19]–[30]. Such procedures include developing effective interaction potentials among residues, exploring the phase space selectively, resorting to efficient and effective methods, etc. In modeling the structure of proteins, knowledge-based contact matrix [11]–[18] is extensively used to develop phenomenological residue-residue interactions. A number of knowledge-based contact potentials based on a growing ensemble of protein data base has been developed to understand the folding dynamics of proteins. We propose a simulated residue-residue interaction based on an all-atom Molecular Dynamics (MD) simulation. Analogous to knowledge-based phenomenological interaction [17], [18], simulated residue-residue interaction matrix can be used as an input to phenomenological interaction in hierarchy to carry out large-scale computer simulations (see below). Such a coarse-grained approach has been recently used to understand the binding of peptides with a graphene substrate [1] where the simulated interaction (residue-substrate) matrix is relatively small. Results of all-atom approach, i.e., the relative binding of each residue are verified by the coarse-grained method before large-scale simulations were performed [1]. In this article, we focus on a residue-residue interaction matrix, which is much larger than the residue-substrate [1] interaction matrix and study the self-assembly of peptides, P1.

As mentioned above, solvent plays an important role in modulating the structure and assembly of peptides and proteins. Understanding the self-assembly of unsolvated peptides (i.e. in vacuum) first is also important before investigating the effect of solvent. A number of neutron scattering experiments are recently performed on powder samples of proteins where simulations in vacuum are used to interpret the scattering data [31]–[33]; these studies also include the effect of solvent by identifying the differences. Thus, apart from the simplicity, there is a value in exploring the structure and dynamics of peptides in vacuum (appropriate for powder samples), i.e., constraining to residue-residue interactions alone before we incorporate the solvent.

## Model and Method

Simulations are carried out in two steps in hierarchy (bottom-up coarse-graining): (*i*) estimate the residue-residue interaction among 20 amino acids (210 interaction pairs of the 20×20 matrix) using an all-atom MD simulation, and (*ii*) use the simulated residue-residue interaction matrix as an input to a phenomenological interaction in the coarse-grained representation of peptide chains (see below).

### All-atom approach

The actual residue interaction inside proteins and between proteins depends on amino acid size, geometry, conformation and the local biochemical environment. It is a very difficult task to develop a portable force field to take all the effects into account. The extreme simplicity of the potential function is based on the hypothesis that a system fluctuates around an equilibrium reference configuration. To find the equilibrium reference configuration between two residues, we resort to molecular dynamics simulation in vacuum using the AMBER ff99SB force field.

A total of 210 residue pairs are simulated in the same protocol using NAMD2.9 simulation software. The initial backbone positions of two residues are the same. The mass centers of backbones are 1 nm apart. Each amino acid is capped by an acetyl beginning group, ACE, and an N-methylamine ending group, NME, to avoid strong terminal interactions and to mimic the bond connectivity. Due to bond connectivity and counter ionic effect in solution, like charged residues can appear side by side in a protein. For charged residues, necessary counter ions are added to neutralize the side chains. Each system is minimized for 2000 steps using a sophisticated conjugate gradient and line search algorithm, then heated up to 300 K increasing 30 K from 0 K every 1000 steps, and then equilibrated for 20 ns in vacuum setting a cutoff distance of 1.2 nm and a switching distance of 1.0 nm for both van der Waals and electrostatic interactions. Finally, another 10 ns production run is performed and trajectories are stored every 10 ps for each system.

The above MD simulations are independently repeated three times for each system. The obtained total system potentials in the last 10 ns for each of the three independent simulations are averaged along their own trajectories and compared. The trajectories corresponding to the lowest average total potential in the last 10 ns are used to calculate the interaction energy between two residues according to the X-PLOR van der Waals function
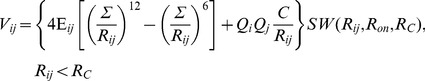
(1)where R_ij_ is the distance between two atoms. Q_i_ and Q_j_ are atomic partial charges. C is a dimensional constant. The cutoff distance R_C_ is equal to1.2 nm. The switching distance R_on_ is 1.0 nm. The switching function SW has the form



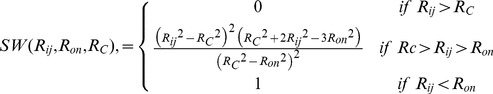
(2)The interaction energy matrix is presented in Table 1. In our simulations, we assume that all residue pairs are exposed to each other. In reality, hydrophobic residues are buried inside a protein, whereas hydrophilic residues are exposed to the environment. We constrain here to residue-residue interactions alone for simplicity; solvent (explicit and implicit) could be incorporated to modulate the distribution of residues (hydrophobic or hydrophilic depending on the nature of solvent) and therefore the structure of the peptide accordingly.

**Table 1:** The minimum pairwise interaction energy (kcal/mol) of 20 amino acids from all-atom MD simulation in vacuum (like charge pair interaction energy, i.e., D-D, D-E, E-E, R-R, R-K and K-K are positive all others are negative).

	A	G	V	L	I	P	F	M	W	C	D	E	R	K	H	N	Q	S	T	Y
A	6																			
G	3	7																		
V	5	7	3																	
L	5	8	4	4																
I	3	4	3	4	6															
P	2	2	2	2	5	1														
F	8	6	4	8	6	4	7													
M	7	8	5	8	5	4	6	8												
W	9	9	9	12	11	11	14	14	15											
C	7	10	4	4	8	9	6	8	8	5										
D	21	23	22	21	21	11	20	27	26	30	67									
E	22	21	21	22	21	15	22	27	11	29	68	78								
R	22	21	27	26	25	27	23	24	30	19	132	138	41							
K	21	23	23	26	25	29	31	6	39	22	138	149	45	43						
H	11	9	−9	−9	8	10	13	14	18	11	29	30	30	29	15					
N	11	14	11	10	9	6	12	14	15	13	22	25	19	24	16	18				
Q	12	12	11	11	10	11	13	12	12	12	37	30	31	30	15	19	23			
S	11	14	9	9	6	3	13	9	16	7	39	34	22	23	14	13	18	13		
T	10	12	6	10	6	3	12	8	12	11	39	39	19	22	17	13	18	12	10	
Y	10	11	7	7	5	9	10	12	11	10	25	24	35	29	14	10	19	11	10	12

### All-residue approach

The intra-molecular detail of each residue is ignored in a coarse-grained description of the peptide, which is a set of nodes tethered together by flexible peptide bonds on a cubic lattice [1]. A residue is represented by a node and its specific characteristics are captured by unique residue-residue interactions (see Table 1). We use a bond-fluctuation method as before [17], [18] to exploit the efficiency of the discrete lattice with ample degrees of freedom. Peptide nodes interact with neighboring nodes with a generalized Lennard-Jones potential,
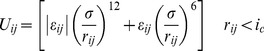
(3)where *r_ij_* is the distance between the residues at site *i* and *j*, *r_c_ = √8* is the range of interaction and *σ = 1* in units of lattice constant. The residue-residue pair interaction (Table 1) is used for the coefficients *ε_ij_* (a measure of the depth) of the generalized LJ potential.

We consider a cubic lattice of size *L^3^*. Peptides (P1) of a volume fraction (*C_p_*) are then placed in the box in random configurations subject to excluded volume constraint. With the constraints on fluctuating bond length *l* (*2 ≤ l≤ √(10)* in units of lattice constant) and excluded volume, each residue performs its stochastic motion with the Metropolis algorithm as follows. An attempt is made to move a randomly selected residue of a randomly selected peptide chain from its current position at site (*i*) to a neighboring site *j* with the Boltzmann probability *exp(−ΔE_ij_/T)*, where *ΔE_ij_* is the change in energy (Eq. 3) between its new (*E_j_*) and old (*E_i_*) configuration *ΔE_ij_  =  E_j_ – E_i_* and *T* is the temperature in reduced units of the Boltzmann constant and the energy (*ε_ij_*). Attempts to move each residue in the simulation box once defines the unit Monte Carlo step (MCS). Simulations are performed for a sufficiently long time to identify the structural changes from small to large scales. A number of local and global physical quantities are evaluated during the simulations including the structural profile of each residue, the variations of the root mean square (RMS) displacement of the center of mass of each peptide and the radius of gyration with the time steps, structure factor and radial distribution function of the self-assembled structures. Simulations are performed at different temperatures at a peptide concentration *C_p_ = 0.1* with as many as *100* independent samples on a *64^3^* lattice to estimate the average value of the physical quantities. We have carried out simulations with different peptide concentrations but we will focus on the low concentration for clarity of the structural evolution with the temperature. Different size lattices are also used to assure that results on the qualitative trends of the physical quantities remain independent of the sample size.

## Results and Discussion

**Figure 1:**
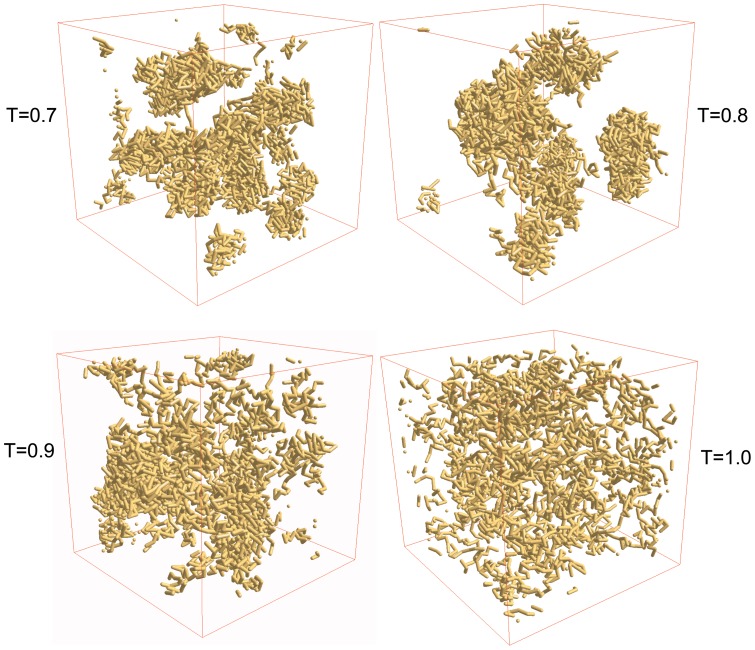
Snapshots of peptides P1 at T = 0.7 (top left), 0.8 (top right), 0.9 (bottom left), 1.0 (bottom right) at the end of 5×10^5^ MCS time on a 64^3^ lattice with peptide concentration *C_p_ = 0.1*.

Peptides (P1) are randomly distributed initially in their random conformations in the simulation box. As residues perform stochastic motion with the Metropolis algorithm, each peptide moves and undergoes conformational changes with the time step. Peptide segments within the range of interaction may be bound due to non-covalent interactions and unbound due to thermal agitation. Distribution of peptides evolves with time and reaches a steady-state configuration. Time to reach the steady-state equilibrium depends on the temperature. Figure 1 shows a set of typical snapshots at the steady state at a range of low-to-high temperatures, which illustrates the differences in morphology due to distribution of peptides. Both, residue-residue interactions and temperature compete in the self-assembly of peptides and their dispersion. At low temperatures (T = 0.7, 0.8), the residue-residue interaction is more dominant over the thermal energy. Peptides self-assemble into aggregates with relatively high density due to attractive residue-residue interactions. Peptides disperse at high temperature (T = 1.0) (overcoming the non-covalent residue-residue interactions) with almost uniform low density throughout the lattice. The aggregation of peptides is interaction-driven at low temperature where system reaches the steady state rather quickly. In fact, at low temperatures some peptides may be trapped very quickly in the self-assembly process without exploring all possible conformations. Peptides explore conformations rather more thoroughly at high temperatures. Consequences of such a difference in thermal response of peptides should be reflected in physical properties of peptides and their self-assembly (see below).

**Figure 2:**
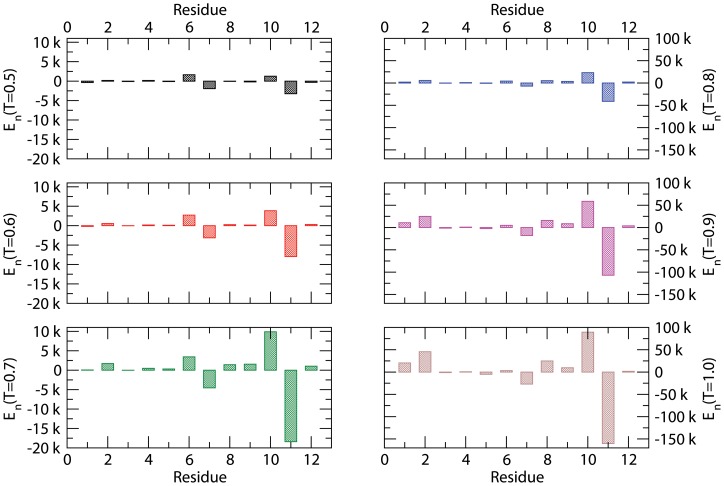
Energy (*E_n_*) of each residue of P1:^ 1^H ^2^S ^3^S ^4^Y ^5^W ^6^Y ^7^A ^8^F ^9^N ^10^N ^11^K ^12^T at temperature *T = 0.5–1.0*. Simulations are performed on a *64^3^* lattice with the peptide concentration *C_p_ = 0.1* with as many as 100 independent samples to estimate the average.

As mentioned above, we have analyzed both local and global physical quantities. The energy profile (energy of each residue in each peptide at equilibrium) of the peptide P1 is presented in Figure 2 at different temperatures (T = 0.5–1.0). We track the energy of each residue and peptide during the course of the simulation. The energy E_n_ (n = 1, 2, …, 12) of each residue node is the average value evaluated from all peptide chains and all independent samples using Eq. 3. The profile pattern remains nearly the same at low and high temperatures albeit with lower and higher energy values. Two residues with the highest and lowest energy are ^10^N and ^11^K, respectively, which shows that not only the specificity of a residue is important but also its sequential position (e.g., compare the energy of ^9^N and ^10^N). The corresponding mobility profile of the peptide (see Figure S1) shows that minimum energy of a residue does not necessarily correspond to lowest mobility. The residues at the ends (^1^H, ^12^T) are relatively more mobile than those in the interior due to constraints imposed by the peptide bonds. Residues ^7^A and ^6^Y appear to be the most mobile and are the second lowest and second highest energy, respectively (see Figure 2). Thus energy alone is not a measure of mobility of a residue in a peptide chain.

**Figure 3:**
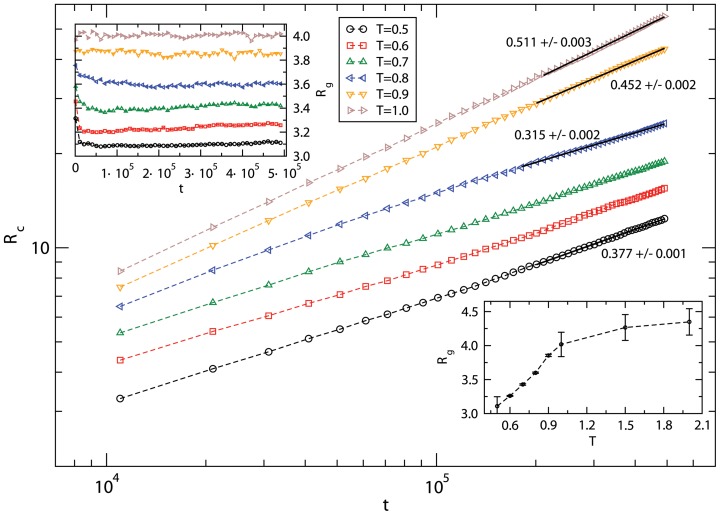
Variation of the root mean square (RMS) displacement of the center of mass of peptides with the time steps on a log-log scale. Inset figures show the variation of the radius of gyration with the time step (top left) and dependence of the equilibrium *R_g_* on the temperature (*T*). Simulations are performed on a *64^3^* lattice with the peptide concentration *C_p_ = 0.1* with as many as 100 independent samples to estimate the average.

**Figure 4:**
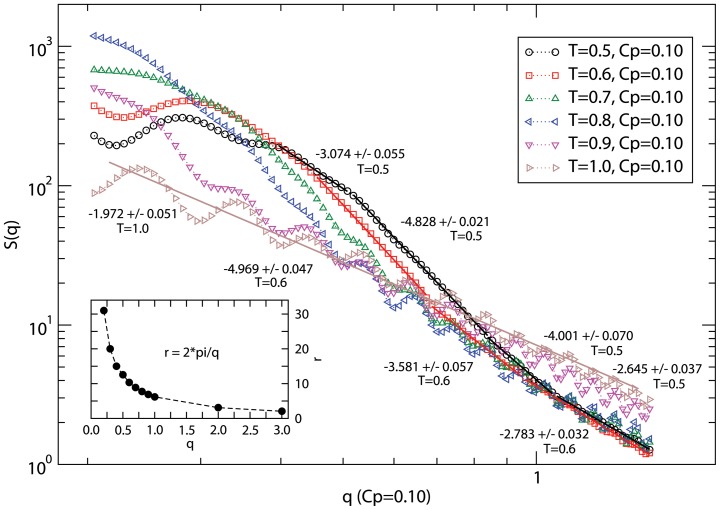
Variation of the structure factor *S(q)* with the wave vector *q* at temperature *T = 0.5–1.0*. A spatial scale of the wave vector q is included in the inset for a guide. Simulations are performed on a *64^3^* lattice with the peptide concentration *C_p_ = 0.1* with as many as 100 independent samples to estimate the average.

Let us examine how peptides move and conform. Figure 3 shows the variation of the average root mean square (RMS) displacement *R_c_* of the center of mass of a peptide chain with the time step (*t*) in temperature range *T = 0.5–1.0*. The asymptotic dependence of *R_c_* on *t* can be described by a power-law, *R_c_* ∞ *t^γ^*, where *γ = ½* describes the diffusive nature of the peptides' motion. At a high temperature (*T = 0.5–1.0*), *γ ≈ ½* (see Figure 4) but *γ (γ ≈ 0.3−0.4) <½* at low temperatures *T = 0.5–0.8*, which implies sub-diffusive dynamics of the peptide chain. The residue-residue interaction dominates over the thermal energy at low temperatures. The peptide chains self-assemble into aggregates on encountering each other. Because of the self-organizing morphology, the mobility of the residues, and therefore the peptide chains, decreases at low temperatures leading to sub-diffusive dynamics. Peptide chains become free and diffuse as residues unbind from assembly at high temperatures. The overall dynamics of peptides is thus consistent with the visual inspection of the snapshots (Figure 1). Variation of the average radius of gyration (*R_g_*) of peptide chains with the time step (inset Figure 3) suggests that the conformations have reached equilibrium during the course of simulation at each temperature. How does the equilibrium value of *R_g_* depend on the temperature? The plot of *R_g_* with the temperature (*T*) is included in Figure 4 (inset). We see that the radius of gyration increases monotonically on raising the temperature in the low-to-intermediate temperature range (*T = 0.5–1.0*) and approaches a constant at high temperatures.

Large-scale structures resulting from the self-assembly of peptides can be studied by examining the radial distribution function (RDF), which is the average number of particles (residues) from the center. We have analyzed (figure S2) the spatial dependence of RDF in the temperature range *T = 0.5–1.5*. We find that the rapid assembly of peptides at low temperature (*T = 0.5*) leads to an aggregate with high density at the center followed by a sharp decay with the distance (*r*). The density of aggregates spreads on raising the temperature (*T = 0.5–0.8*). Peptide chains disperse at high temperature where no aggregate develops.

The multi-scale morphology of the self-assembly of peptides can be studied by analyzing the structure factor *S(q)* (Figure 4),
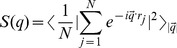
(4)where *r_j_* is the position of each residue and *|q| = 2π/λ* is the wave vector of wavelength, *λ*. One can study the mass distribution of particles (residues) by estimating the exponent *ν* in the power-law scaling *S(q) ∝ q* ^−*1/ν*^. Spatial scaling of mass (*M*) with the radius of gryation (*R_a_* ≈ *λ*) of the aggregate, *M ∝ R_a_^D^* provides an estimate of its effective dimension *D = 1/ν*. Higher value of *D*, e.g., *D = 3* implies a solid while *D = 2* represents an ideal chain with a heterogeneous mass distribution on a cubic lattice; *D>3* does not make sense and could be an artifact of fitting data in the wrong regions including crossover regimes.

Variation of the structure factor *S(q)* with *q* provides an insight into a rather rich structure at all length scales. Note that the wave length *λ*≈*5–15* (in units of lattice constant) is comparable to spatial spread of a peptide aggregate and corresponds to wave vector *q* ≈ *0.5–1.0*; the radius of gyration of peptide *R_g_≈3.2–4.2* (see figure 3). At the low temperature (*T = 0.5*), we see a rather solid morphology (*q*≈*0.4–0.8*). Spreading of the solid-like morphology of self-assemly of peptides is clearly seen on raising the temperature (*T = 0.5, 0.6*). Oscillation in *S(q)* sets at smaller scales (order of the chain length) at higher temperature (*T = 0.8*) becomes persistent at all length scales at high temperatures (*T≥ 1.0*) with dispersion of peptide chains. We would like to point out that the oscillatory nature of the structure factor is not an artifact of cubic lattice considered here but due to thermodynamics of peptides at high temperatures. A general fit of oscillatory data at *T = 1.0* shows that the mass distribution of peptide is heterogeneous in morphology of a random walk. Thus, the structural evolution of peptides, from a highly dispersed peptide chains at high temperatures (an expected thermodynamic behavior) to its aggregation on reducing the temperature can be studied by such hierarchical coarse-grained approach. Unfortunately, we are not aware of experimental data on such assembly at present.

**Figure 5:**
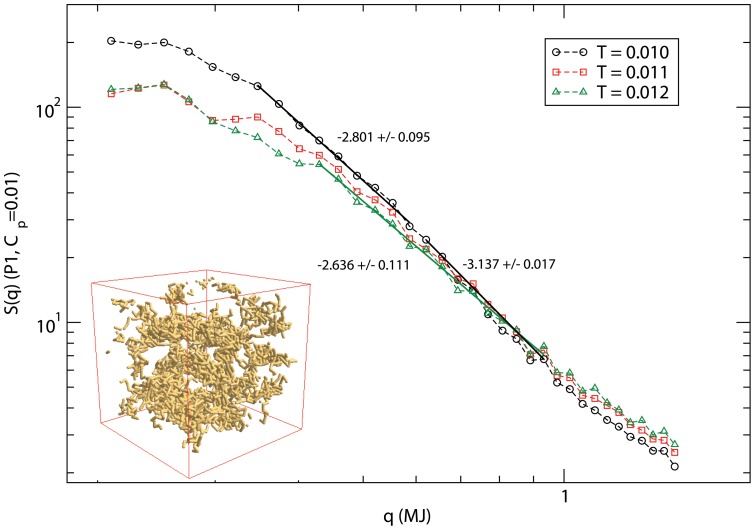
Variation of the structure factor *S(q)* with the wave vector *q* at temperature *T = 0.010, 0.011, 0.012*. Miyazawa-Jernigan (MJ) [12] residue-residue interaction is used for the coefficient *ε_ij_* in coarse-grained potential (eq. 3). Simulations are performed on a *64^3^* lattice with the peptide concentration *C_p_ = 0.1* with as many as 100 independent samples to estimate the average. Inset is a snapshot at T = 0.010.

Alternatively, one may consider other interactions such as knowledge-based interaction (previously used in study of protein folding [17], [18]) which is derived from the ensemble of protein structures available in the protein data bank (PDB). X-ray crystallographic data in PDB represent snapshots of proteins' conformations in their native structures in unique solvent. Knowledge-based analysis [12] involves further assumptions and approximations to derive residue-residue contact maps among the residues. Thus, the knowledge-based interaction captures the essence of residue-residue interaction in a different environment which is much more complex than the simulated residue-residue interaction considered here. This does not mean that the knowledge-based interaction is superior than the simulated one as the prior method resorts to a number of assumptions and approximations. Nevertheless it is worth exploring what happens if we use the knowledge-based residue-residue interactions (previously used in study of protein folding [17], [18]) in place of simulated interaction? We have carried out such simulation with the classic MJ interaction matrix [12]. Data for the variation of the structure factor *S(q)* with *q* is presented in figure 5. Note that the temperature scales are dramatically different from those used with simulated residue-residue interaction matrix. This is primarily due to differences in magnitude of the MJ matrix elements [12] and the simulated interaction (table 1). We see that peptide chains assemble into aggregates with a rather solid density (*D*≈*3*) at the low temperature (*T = 0.010*) which shows a dispesrive trend of peptides on increasing the temperature (e.g. *D*≈*2.6* at *T = 0.012*). Despite the shift in temperature scale, the general self-organizing trend (from aggregation to dispersion) appears consistent with our hierarchical coarse-grained approach. Because of the differences in assumptions and approximation made in deriving the knowledge-based contact matrix [12] and the direct simulation of invidual residue-residue interaction (i.e. with free residue unlike the residues as a part of protein), quantitative differences (figure 4 and 5) are not un-expected. Such coarse-grained approach with specific residue-residue interaction matrix provides an additional alternative to address complex problems in bio-inspired assembly.

## Conclusions

A hierarchical coarse-graining scheme introduced here thus provides a useful method to investigate multi-scale self-organizing structures of such complex constituents as peptides (e.g. P1). It involves all-atom MD simulations to estimate the residue-residue interactions in which twenty amino acids constitute 210 independent pairs, each of which has its unique interaction energy (Table 1). The simulated interaction matrix forms the basis for the residue-residue interaction similar to knowledge-based contact matrices [12]–[18] in an all-residue representation of the peptide chain. The structural evolutions are analyzed in detail by examining both local and global physical quantities spanning the entire scale.

Visualizations of the self-organizing assembly clearly show a systematic change in morphology at a range of length scales with the temperature. We find that the energy profile of residues does not necessarily dictate their mobility, which would have been expected for a simple system with its thermodynamics controlled primarily by interactions. Spatial distribution of residues within a peptide does respond to self-assembly of peptides. The dynamics of peptides as they perform their stochastic motion during the self-assembly and their dispersion shows distinct but appropriate characteristics, i.e., sub-diffusion and diffusion. The radius of gyration of peptides responds linearly to temperature as the self-organized aggregates expand on raising the temperature before approaching saturation at high temperatures. The spatial variation of the radial distribution function reveals that the solid core of the aggregates softens as the density spreads with the temperature. The scaling of the structure factor with the wave vector provides valuable insight into the multi-scale structure of the assembly. For example, on raising the temperature from its low value, the relatively high density of the self-assembly expands over the length scale, which is sensitive to temperature, before reaching a well-dispersed distribution of an ideal chain at high temperature. We hope that this study will stimulate experiments with multi-scale resolution to verify or contradict our predictions. Such a hierarchical coarse-graining is not limited to Monte Carlo simulations with bond-fluctuation methods (used here) but could be extended to such approach as Molecular Dynamics.

As mentioned in the beginning, interaction of peptides in a solvent matrix (implicit or explicit) plays a critical role in modulating the structure and dynamics of peptides and its assembly. For example, we have shown [23], how the structure and dynamics of a protein is affected by the quality of solvent by incorporating the hydrophobicity of each amino acids and its unique interaction in an effective solvent medium. Structure of a histone is found to exhibit a non-monotonic response to solvent quality in a recent Monte Carlo simulation [34]. There are enormous opportunities to improve the simulated residue-residue interaction matrix as well as in incorporating such realistic factors as solvent and substrate, some of which may be taken up in future efforts.

## Supporting Information

Figure S1 Mobility (*M_n_*: average number of successful hops) of each residue of P1:^ 1^H ^2^S ^3^S ^4^Y ^5^W ^6^Y ^7^A ^8^F ^9^N ^10^N ^11^K ^12^T at temperature *T = 0.5–1.0*. Simulations are performed on a *64^3^* lattice with the peptide concentration *C_p_ = 0.1* with as many as 100 independent samples to estimate the average.
(TIF)

Figure S2 Variation of the radial distribution function (RDF), a measure of the number of particles (residues) with distance *r* at temperature *T = 0.5–1.5*. Simulations are performed on a *64^3^* lattice with the peptide concentration *C_p_ = 0.1* with as many as 100 independent samples to estimate the average.
(TIF)
